# Ongoing Health Care Expenditure in Survivors of Sepsis in The Intensive Care Unit

**DOI:** 10.1186/2197-425X-3-S1-A21

**Published:** 2015-10-01

**Authors:** M Koster-Brouwer, K van de Groep, P Klein Klouwenberg, W Pasma, T van der Poll, M Bonten, O Cremer

**Affiliations:** University Medical Center Utrecht, Intensive Care Center, Utrecht, Netherlands; University Medical Center Utrecht, Julius Center for Health Sciences and Primary Care, Utrecht, Netherlands; University Medical Center Utrecht, Medical Microbiology, Utrecht, Netherlands; Academic Medical Center, University of Amsterdam, Center of Experimental and Molecular Medicine, Amsterdam, Netherlands; Academic Medical Center, Infectious Diseases, Amsterdam, Netherlands

## Introduction

Direct costs associated with an intensive care unit (ICU) admission for sepsis are approximately €30,000.[1] However, total cost for society is likely to be much higher, because survivors of sepsis may suffer from long-term sequelae that generate ongoing need for health care resources.[2]

## Objectives

To estimate the difference in annual health care expenditure before and after an ICU admission for sepsis.

## Methods

Data were derived from a prospective cohort study in two tertiary ICUs in the Netherlands in 2011 and 2012. Patients were included if they had survived one year following a sepsis episode in the ICU. Health care consumption and reimbursed costs were derived from a database of a Dutch health insurance company. The medical ethics committee of the UMC Utrecht approved the study and waived the need for informed consent (IRB-number 14-095).

## Results

**Figure 1:**
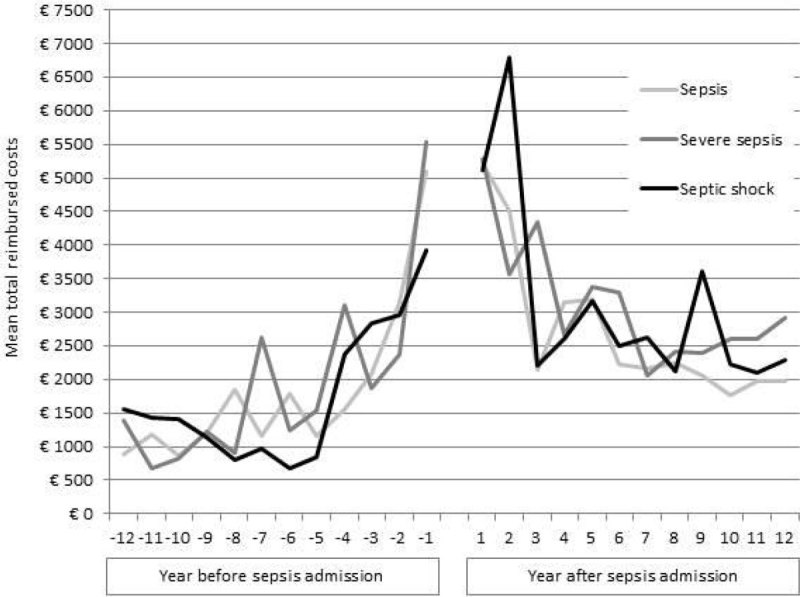
Figure 1

Of the 396 eligible patients, for 21 (5.3%) there was no information on costs available in the study period, leaving 375 subjects for analysis. An overview of reimbursed costs is given in table 1. For each stage of sepsis severity, total costs were significantly higher in the year following a sepsis admission compared to the year before (p < 0.001 for all stages, figure 1). This overall increase in costs was due to the increased use of long-term (home) care (p < 0.001 for all stages), and consultations of the general practitioner, paramedic, or mental health professional (p < 0.001 for sepsis, p = 0.008 for severe sepsis, and p < 0.001 for septic shock). in the year after the sepsis episode more patients (46%) resided in a long-term care facility or received home care than before the event (10%). Likewise, the proportion of patients receiving paramedical and mental health care increased from respectively 26% to 34% and from 3% to 9%. Hospital costs following the sepsis episode were significantly higher for patients who had septic shock during their ICU stay (p = 0.038), but not for patients who had sepsis (p = 0.436) or severe sepsis (p = 0.292). The opposite was seen for drug use, with a significant increase in costs for patients who had sepsis (p = 0.002) or severe sepsis (p = 0.021), and a non-significant increase for patients who had shock (p = 0.167). We observed no differences in total health care expenditure by disease severity (p = 0.323).

**Figure 2:**
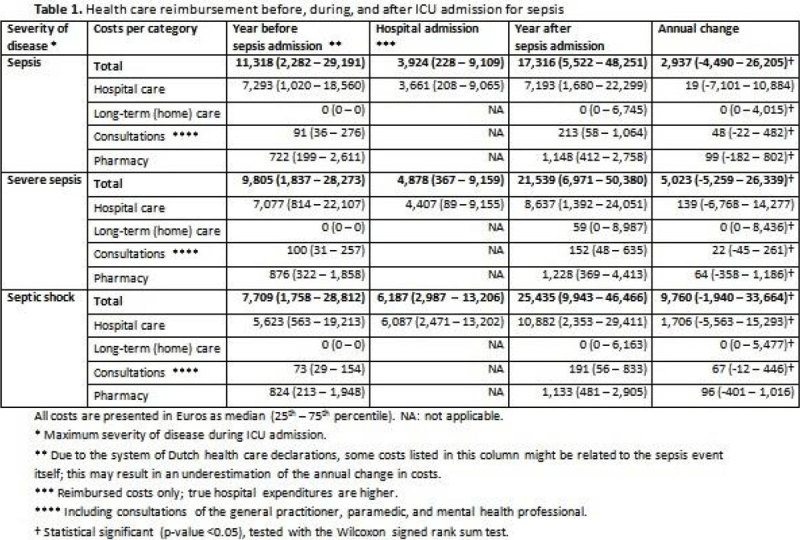
Figure 2

## Conclusions

After successful initial ICU treatment, survivors of sepsis generate substantial health care costs in the year following admission.

## Grant Acknowledgment

This work was supported by the Center for Translational Molecular Medicine, project MARS (grant 04I-201). MB received research funding from the Netherlands Organization of Scientific Research (NWO Vici 918.76.611).
